# Cell envelope defects of different capsule‐null mutants in K1 hypervirulent *Klebsiella pneumoniae* can affect bacterial pathogenesis

**DOI:** 10.1111/mmi.14447

**Published:** 2020-01-20

**Authors:** Yi Han Tan, Yahua Chen, Wilson H. W. Chu, Lok‐To Sham, Yunn‐Hwen Gan

**Affiliations:** ^1^ Department of Biochemistry Yong Loo Lin School of Medicine, National University of Singapore Singapore; ^2^ Department of Microbiology and Immunology Yong Loo Lin School of Medicine, National University of Singapore Singapore

**Keywords:** bile salt resistance, capsule, cell envelope defects, hypervirulent, intestinal colonisation, *Klebsiella pneumoniae*

## Abstract

Hypervirulent *Klebsiella pneumoniae* (hvKP) causes *Klebsiella*‐induced liver abscess. Capsule is important for the pathogenesis of *Klebsiella* in systemic infection, but its role in gut colonisation is not well understood. By generating Δ*wcaJ*, Δ*wza* and Δ*wzy* capsule‐null mutants in a prototypical K1 hypervirulent isolate, we show that inactivation of *wza* (capsule exportase) and *wzy* (capsule polymerase) confer cell envelope defects in addition to capsule loss, making them susceptible to bile salts and detergent stress. Bile salt resistance is restored when the initial glycosyltransferase *wcaJ* was inactivated together with *wzy*, indicating that build‐up of capsule intermediates contribute to cell envelope defects. Mouse gut colonisation competition assays show that the capsule and its regulator RmpA were not required for hvKP to persist in the gut, although initial colonisation was decreased in the mutants. Both Δ*rmpA* and Δ*wcaJ* mutants gradually outcompeted the wild type in the gut, whereas Δ*wza* and Δ*wzy* mutants were less fit than wild type. Together, our results advise caution in using the right capsule‐null mutant for determination of capsule's role in bacterial pathogenesis. With the use of Δ*wcaJ* mutant, we found that although the capsule is important for bacterial survival outside the gut environment, it imposes a fitness cost in the gut.

## Graphical abstract

The capsule of hypervirulent *Klebsiella pneumoniae* is important for causing severe disease. Hypervirulent *K. pneumoniae* colonises the gut and is thought to circulate in the population by the faecal–oral route. However, the use of different mutants defective in capsule production cause confusion as we found that some mutants have additional cell envelope stability issues besides not having a capsule that make interpretations on their contribution to disease difficult. We show that using the appropriate capsule null mutant, capsule is important for causing systemic disease but not intestinal colonisation.

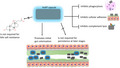

## 1 INTRODUCTION

Community‐acquired, hypervirulent *Klebsiella pneumoniae* (hvKP) has become the leading cause of microbial‐associated pyogenic liver abscess (PLA) in Singapore, Taiwan and other parts of Asia (Alsaif, Sudhakar, Chan, & Archuleta, 2011; Bilal, Volz, Schneider, Fideler, & Podschun, 2014; Lo et al., 2015; Siu, Yeh, Lin, Fung, & Chang, 2012; Wang et al., 1998). HvKP typically possesses a thick, hypermucoid K1 or K2 capsule and colonies on agar give a positive string test (Li, Zhao, Liu, Chen, & Zhou, 2014; Shon, Bajwa, & Russo, 2013). HvKP likely circulates in the population via the faecal–oral route (Chung et al., 2012; Siu et al., 2011) and colonises the gut of both healthy and susceptible individuals. In more susceptible individuals, such as diabetic patients, hvKP disseminates from the gut to the bloodstream and results in liver abscess and bacteremia (Fung et al., 2012; Kontopoulou et al., 2019). A recent analysis has shown that over 80% of liver abscess isolates belong to a CG23 group 1 sublineage that has disseminated globally in the human population (Lam et al., 2018). This sublineage is distinguished by the presence of a genomic island encoding colibactin (ICEKp10) and the loss of function mutations in the fimbrial protein KpcC and ethanolamine Eat population (Lam et al., 2018). All CG23 group 1 isolates also possess the K1 capsule.

The capsule is a key virulence factor of hvKP and is important for resistance to complement (Alvarez, Merino, Tomas, Benedi, & Alberti, 2000; Cortés et al., 2002; Tan, Gamage, & Gan, 2017), dissemination to the liver (Fang, Chuang, Shun, Chang, & Wang, 2004) and during systemic infection (Cheng et al. 2010; Ho et al. 2011; Palacios et al. 2018; Yeh et al. 2007, 2016). The bacterial capsule could potentially protect hvKP from the assault of the antimicrobial peptides such as cathelicidins, defensins and bile salts present in colonic mucus (Antoni et al., 2013; Campos et al., 2004). The capsule may also aid in the formation of *K. pneumoniae* biofilms, which are more resistant to factors such as to antibiotics and starvation (Wu et al., 2011; Zheng et al., 2018).

The production of the K1 capsule in hvKP is mediated by the Wzx/Wzy‐dependent pathway (Ho et al., 2011; Pan et al., 2015). Briefly, the initial glycosyltransferase WcaJ links the first glucose moiety to the universal lipid carrier undecaprenyl phosphate (Und‐P). Fucose and uronic acid are subsequently added to the nonreducing end of the glucosyl‐P‐Und to synthesise the trisaccharide repeating unit (Ho et al., 2011; Pan et al., 2015). The lipid‐linked intermediate is then flipped across the cytoplasmic membrane, presumably mediated by the transporter Wzx, and then polymerised by Wzy (Whitfield, 2010). Finally, the completed polymer is exported through the pore formed by the secretin Wza and displayed on the surface of the hvKP outer membrane (Li et al., 2014; Whitfield, 2006).

Blocking late steps of the Wzy‐dependent pathway is often lethal to the cell. If the cells survive, they are usually associated with cell shape defects. This phenomenon is likely due to the sequestration of Und‐P (Jorgenson, Kannan, Laubacher, & Young, 2017), which eventually blocks the synthesis of cell wall peptidoglycan. Simultaneously inactivating the initial glycosyltransferase alleviates the cell shape and envelope defects, possibly by restoring the Und‐P lipid carrier pool. To investigate the role of capsule in virulence, one should therefore inactivate the initial glycosyltransferase in the pathway in order to avoid any unwanted deleterious effects on the biogenesis of the cell envelope layer (Manat et al., 2014).

By contrast, studies demonstrating the importance of the K1 capsule in pathogenesis of hvKP were mostly done in a ∆*wzy* deletion genetic background (formerly known as the ∆*magA* mutant, which stands for the **m**ucoviscosity **a**ssociated **g**ene **A**) (Fang et al., 2004; Fang, Lai, Yi, Hsueh, & Liu, 2010; Wu et al., 2008). It is unclear if the ∆*wzy* deletion in hvKP causes secondary cell shape defects, which were observed in many other bacterial species (D'Elia et al., 2006; Jorgenson et al., 2017; Jorgenson & Young, 2016; Sham, Zheng, Yakhnina, Kruse, & Bernhardt, 2018; Xayarath & Yother, 2007). If this is the case, the phenotypes exhibited by the ∆*wzy* mutant must be interpreted with caution.

In this work, we use a K1 clinical isolate SGH10 that has been designated as the reference strain for the CG23 group 1 lineage for our study (Lam et al., 2018). We generated mutants that block different steps in capsule synthesis, regulation and export such as *wcaJ*, *wzy*, *wza*, as well as **r**egulator of **m**ucoidy **p**henotype A (*rmpA*)*.* In addition to the lack of capsule production, *wzy* and *wza* mutants show severe defects in cell envelope stability, which complicates the interpretations of their phenotypes in any infection models. This phenotype can be rescued by inactivation of *wcaJ*, showing that the defects are likely due to the sequestration of Und‐P. We reasoned that results generated from the *wcaJ* mutant are more representative of the effect of losing the capsule in hvKP as this mutant does not demonstrate cell envelope stability issues. Using this mutant in an in vivo competition assay, we demonstrated that the K1 capsule has no detectable role in colonisation persistence in the gastrointestinal tract but is necessary for bacterial pathogenesis during systemic infection.

## 2 RESULTS

### 2.1 Deletions of *wcaJ*, *wza* and *wzy* abolish capsule production

**Figure 1:**
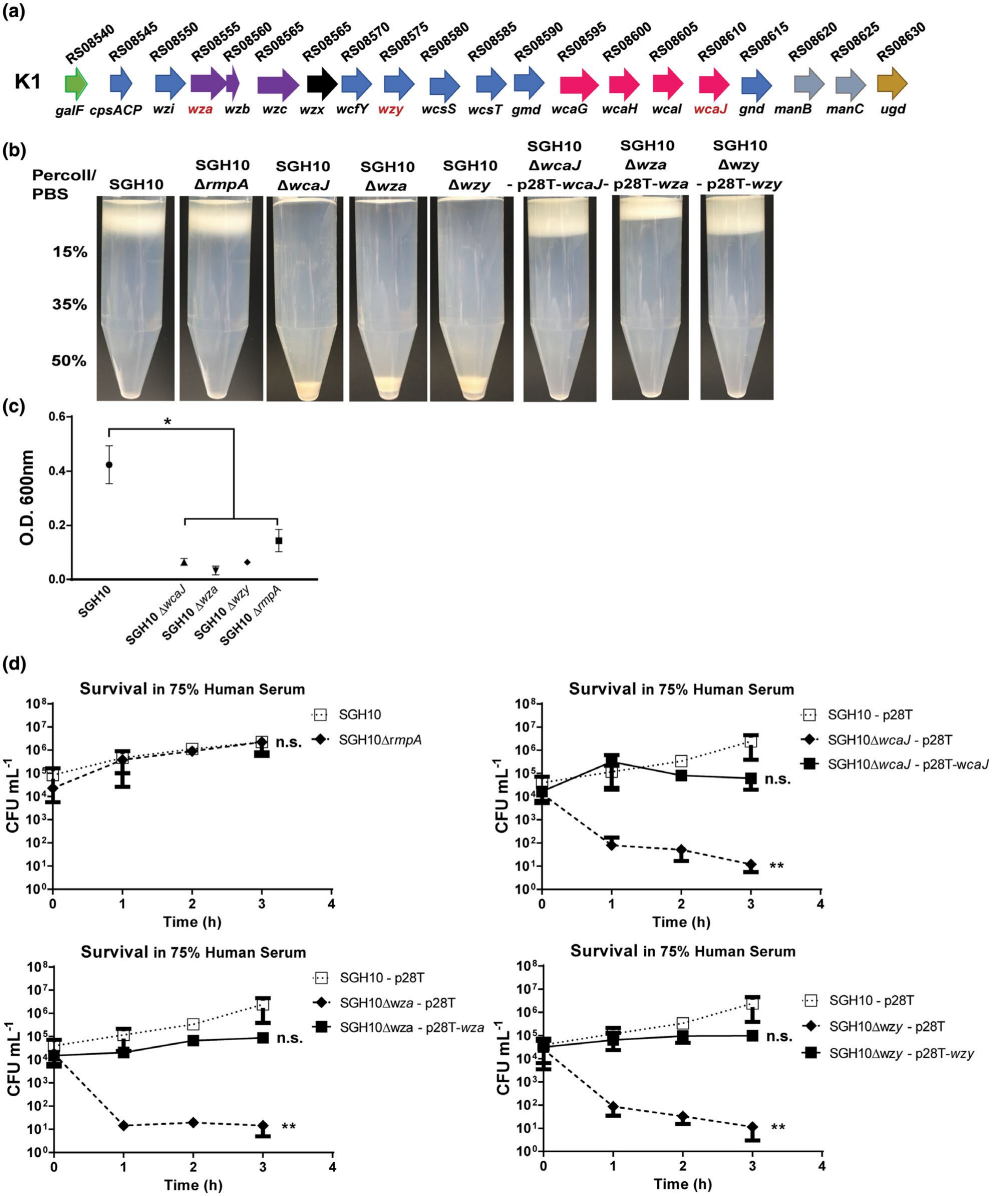
Capsule production of hvKP SGH10. The capsule regulator mutant ∆*rmpA*, and the capsule‐null mutants ∆*wza*, ∆*wzy* and ∆*wcaJ* were created and the mutants were complemented by the expression of the corresponding gene in a pUCP28T(p28T) backbone. (a) The capsule gene cluster of SGH10 (b) Capsule production in stationary phase bacteria SGH10 and the above mutants was measured semi‐quantitatively by density‐dependent separation on a Percoll gradient. (c) Strain mucoidy was measured by low‐speed centrifugation of bacteria in LB. The optical density at 600 nm (O.D. 600) of the supernatant above pellet was measured, and mean ± *SD* are plotted (*n* = 3) and * denotes *p* < .05, while n.s. denotes not significant. (d) Capsule mutants and their complemented counterparts were grown in 75% human serum to determine if they were resistant to complement mediated killing. Mean ± *SD* are plotted (*n* = 3) and Dunnett's multiple comparison test was conducted for CFU values relative to SGH10 at 3 hr. * denotes *p* < .05, ** denotes *p* < .001, while n.s. denotes not significant [Colour figure can be viewed at wileyonlinelibrary.com]

SGH10 belongs to serotype K1 and produces a thick capsule (Lam et al., 2018; Lee et al., 2016). The organisation of the capsule locus in SGH10 is illustrated in Figure 1a. SGH10 carries two copies of *rmpA* on the large virulent plasmid, *rmpA* and *rmpA2*. Plasmid *rmpA2* is truncated and therefore unlikely to be a functional regulator of capsule synthesis, whereas *rmpA* is likely to be the active copy. We examined whether the amount of capsule produced was altered using the Percoll density gradient centrifugation assay. Production of the K1 capsule increases the buoyancy of the cells in Percoll and therefore encapsulated bacteria are retained at the top of the centrifuge tube (Figure 1b, SGH10 lane). In contrast, deletion mutants of *wcaJ*, *wza* and *wzy* exhibited a significantly impaired capsule synthesis. Ectopic expression of *wcaJ*, *wza* and *wzy* in the shuttle vector pUCP28T rescued the capsule‐null phenotype in the corresponding mutant, indicating the deletions had no polar effects.

In line with the previous report (Hsu et al., 2011), deletion of *rmpA* did not alter the amount of capsule significantly, as there was no detectable effect on the cell buoyancy in Percoll (Figure 1b, SGH10∆*rmpA*). However, we noticed that strain SGH10∆*rmpA* did not produce a positive string test. When centrifuged, cells of ∆*rmpA* produced a clearer supernatant compared to wild‐type bacteria (Figure 1c).

Next, we examined whether the deletions of *rmpA*, *wcaJ*, *wza* and *wzy* alter the sensitivity to human serum. As expected, ∆*rmpA* did not show any change in serum sensitivity (Figure 1d). Unencapsulated mutants (∆*wcaJ*, ∆*wza* and ∆*wzy*) were readily killed by serum, but grew in heat‐treated serum (Figure S1). Serum resistance in the capsule‐null strains was restored by complementation with the plasmids harbouring the corresponding capsule genes. We conclude that the ∆*rmpA* deletion has no effect on capsule production while inactivation of *wcaJ*, *wza* and *wzy* abolished capsule synthesis. However, *rmpA* deletion affects the mucoviscosity of hvKP capsule.

### 2.2 hvKP capsule impedes phagocytosis and adhesion

**Figure 2:**
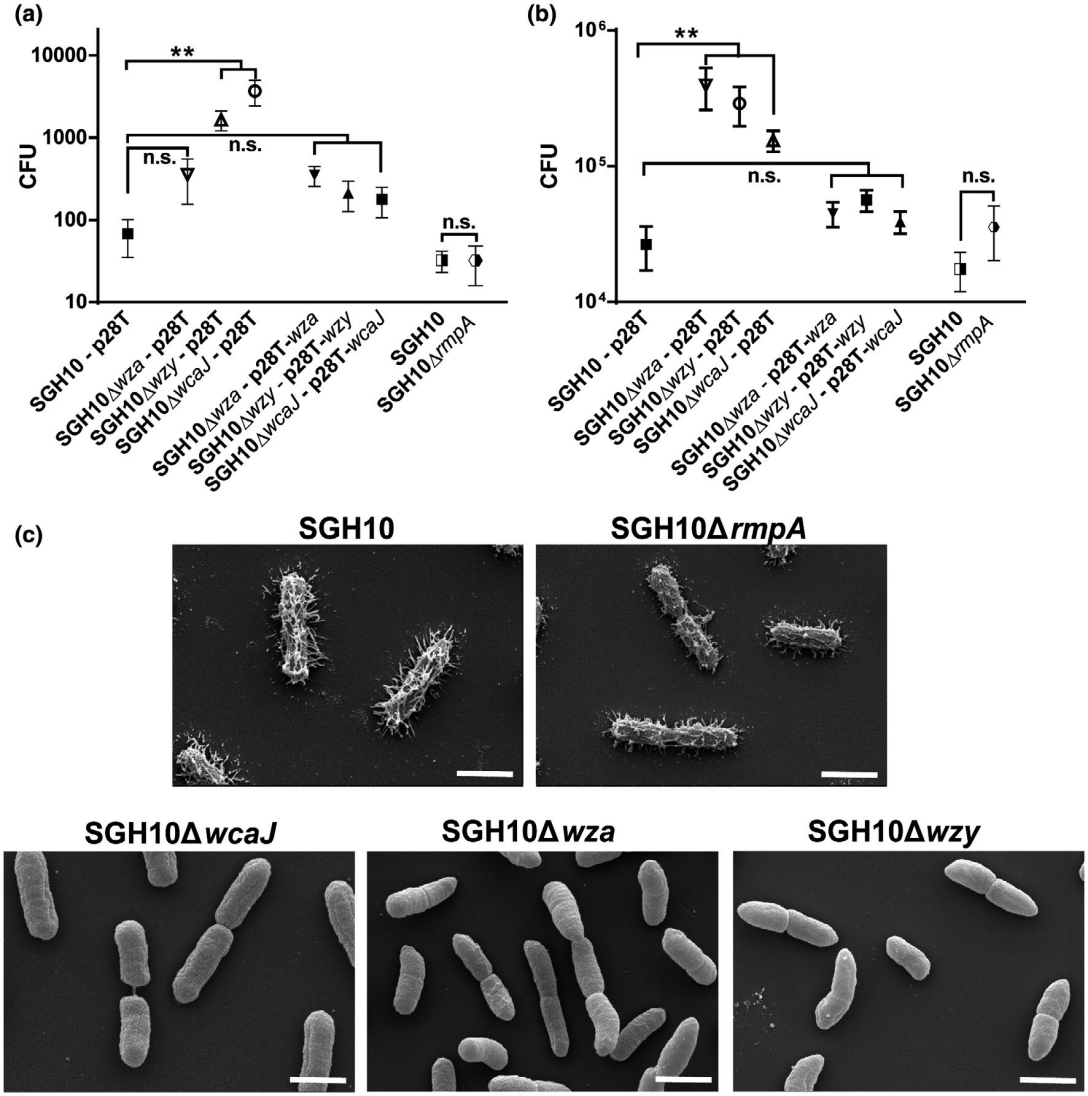
Effect of capsule on association with phagocytic and nonphagocytic cells. (a) Uptake of SGH10, SGH10∆*rmpA*, SGH10∆*wza*, SGH10∆*wzy,* SGH10∆*wcaJ* and the complemented mutants by RAW264.7 macrophages at 2 hr post infection. (b) Adhesion of SGH10 and capsule mutants, as well as the complemented capsule mutants to Caco‐2 cells at 30 min post infection. Mean ± *SD* are plotted (*n* = 3). * denotes *p* < .05 and ** denotes *p* < .001, while n.s. denotes not significant. (c) Scanning electron microscopy images of fixed, log‐phase cultures of SGH10, SGH10∆*rmpA*, SGH10∆*wcaJ*, SGH10∆*wza* and SGH10∆*wzy* cells. The scale bar is 1 μm

Previous studies have demonstrated that *K. pneumoniae* capsule is essential for resistance to phagocytosis (Fang et al., 2004; Yeh et al., 2010). In concordance, ∆*wcaJ* and ∆*wzy* were susceptible to uptake by RAW 264.7 macrophages and their complemented counterparts regained resistance (Figure 2a). However, ∆*rmpA* was phagocytosed at a similar rate as wild type. Intriguingly, the ∆*wza* mutant was more resistant to phagocytosis compared to other capsule‐null mutants, suggesting that the capsule production, but not its export, is required for resistance of phagocytosis.

The ability of bacteria to adhere to the intestines contributes to persistence in the gut. Although one study suggests that the *K. pneumoniae* capsule could decrease adhesion to ileocecal and bladder epithelial cells (Sahly et al., 2000), other studies have shown that *K. pneumoniae* capsule could enhance association to lung epithelial cells and intestinal epithelial cell lines (Clements et al. 2008; Favre‐Bonte, Joly, & Forestier, 1999). The capsule‐null mutants SGH10∆*wcaJ*, SGH10∆*wza* and SGH10∆*wzy* demonstrated significantly increased adhesion to Caco‐2 cells, while SGH10∆*rmpA* did not (Figure 2b). Complementation of the capsule‐null mutants decreased adhesion to that of wild‐type bacteria.

### 2.3 ∆*wza* and ∆*wzy* mutants exhibit cell envelope defects

Scanning electron microscopy (*SEM*) reveals that the capsule of SGH10 spreads out from the surface in multiple directions. Likewise, the SGH10∆*rmpA* mutant is coated with capsule (Figure 2c). SGH10∆*wcaJ*, SGH10∆*wza* and SGH10∆*wzy* cells do not possess the layer of capsular polysaccharide evident on the surface of wild‐type bacteria. Strikingly, while the wild‐type and SGH10∆*wcaJ* cells are rod shaped, SGH10∆*wza* and SGH10∆*wzy* cells exhibit morphological defects. This morphology suggests that their cell envelopes are compromised. The ∆*wza* and ∆*wzy* cells are curved, tapered and thinner at the ends and do not seem to be evenly rod shaped (Figure 2c). When visualised using the FITC dextran exclusion assay, the wild‐type and ∆*rmpA* bacteria were surrounded by a grey halo which indicates the presence of capsule, whereas the capsule‐null mutants did not, further supporting the loss of capsule (Figure S2).

As bacteria progress down the GI tract, they encounter two main stressors, pH and bile stress. Successful pathogenesis requires hvKP to survive under the low‐pH environment of the stomach before traversing the intestines, where the pH is relatively higher. The capsule mutants did not exhibit differences in survival relative to the wild type at pH 4, which is the pH of the mouse stomach (Figure S3), indicating that capsule or the cell shape defects do not affect survival in pH stresses.

**Figure 3:**
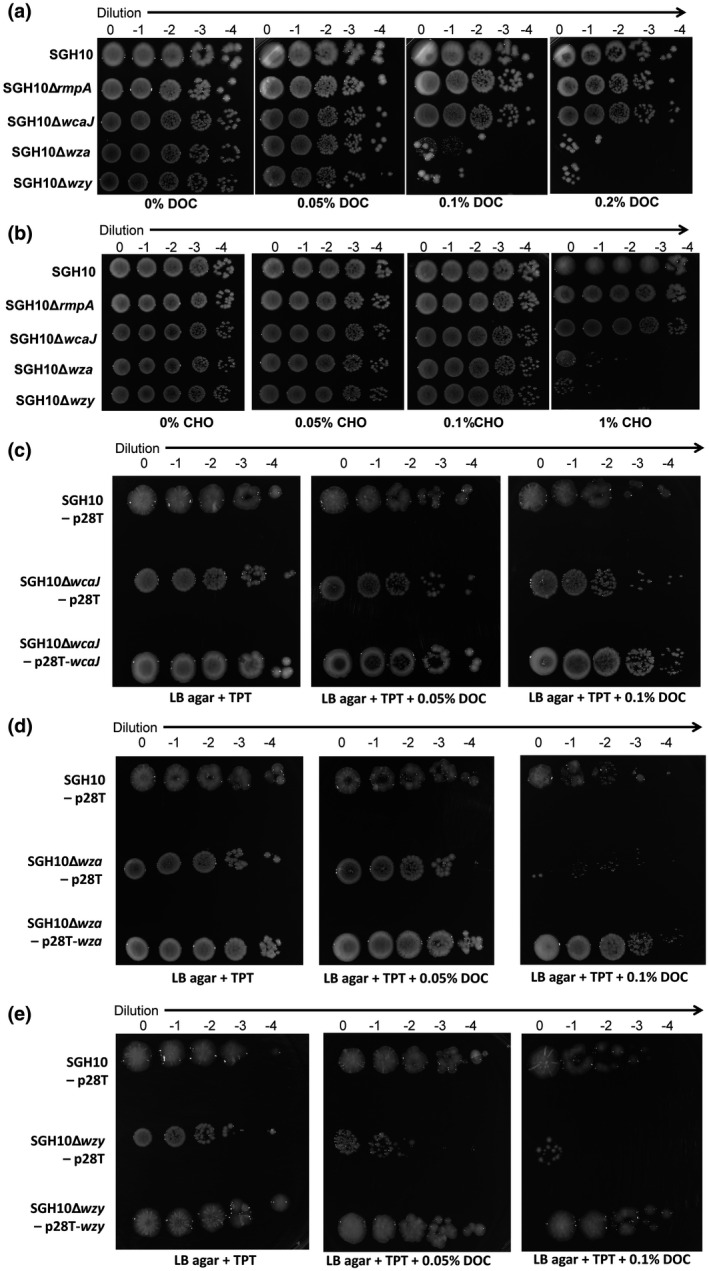
Role of capsule in bile salt resistance. SGH10 and capsule mutants were plated on LB agar supplemented with increasing concentrations of (a) secondary bile salt, sodium deoxycholate (DOC) and (b) primary bile salt, sodium cholate (CHO). Experiments were repeated (*n* = 3), with similar results. (c) Resistance to bile salt is not affected by complementation in ∆*wcaJ* mutants but was restored by complementation in (d) ∆*wza* and (e) ∆*wzy* mutants. SGH10 and complemented capsule mutants were plated on LB agar supplemented with trimethoprim (TPT) and increasing concentrations of DOC

Bile salts are known to modulate the gut microbiome and have a bactericidal effect by disrupting bacterial membrane even below their critical micelle concentrations (Merritt & Donaldson, 2009; Urdaneta & Casadesús, 2017). Moreover, hvKP causes *Klebsiella‐*associated liver abscess and would hence have to be resistant to bile salts in the liver. When SGH10∆*wza* and SGH10∆*wzy* were plated on LB agar supplemented with the secondary bile salt sodium deoxycholate (DOC) at concentrations approximating the physiological concentration of 0.1% in the gut, SGH10∆*wza* and SGH10∆*wzy* exhibited a 100‐fold difference in survival compared to the wild‐type SGH10 (Figure 3a), which suggests that these strains possess an impaired membrane integrity phenotype. SGH10∆*wcaJ* and SGH10∆*rmpA* did not exhibit a difference in survival on DOC relative to wild type (Figure 3a), indicating that the capsule does not affect DOC resistance. The results are similar when the experiment was repeated with the primary bile salt sodium cholate at a concentration of 1% (Figure 3b). Bile salt resistance can be restored with complementation in the ∆*wza* and ∆*wzy* mutants (Figure 3d,e).

**Figure 4:**
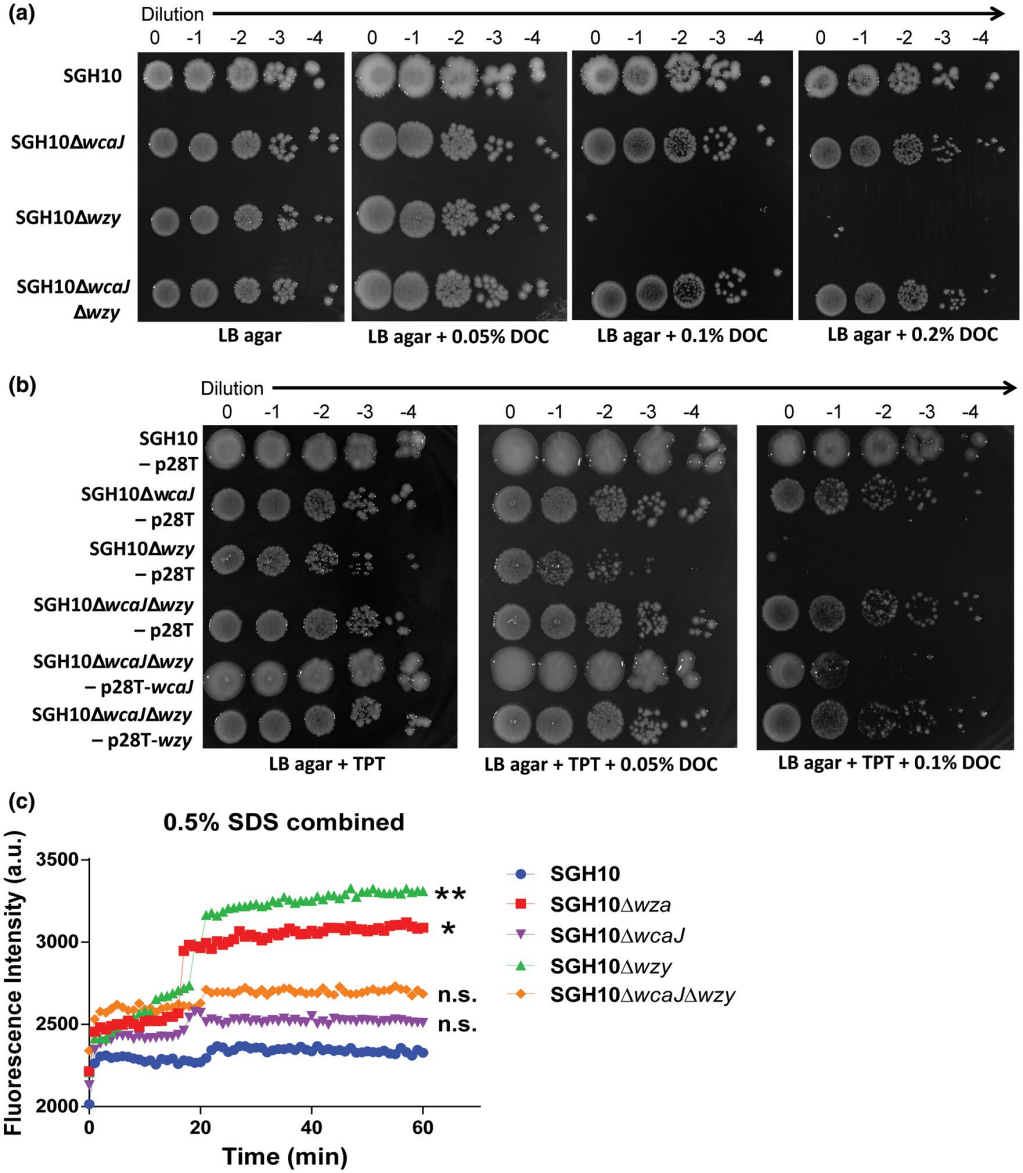
Resistance to bile salt can be rescued in ∆*wzy* mutant through inactivation of *wcaJ*. SGH10 and complemented capsule mutants were plated on LB agar supplemented with trimethoprim (TPT) and increasing concentrations of DOC. (a) Bile salt resistance of the ∆*wcaJ/wzy* mutant compared to the single mutant (b) the ∆*wcaJ/wzy* mutant complemented with *wcaJ* or *wzy*. Experiments were repeated (*n* = 3), with similar results. (c) Propidium iodide accumulation of SGH10 and capsule mutants during sodium dodecyl sulfate (SDS) stress. Mean ± *SD* are plotted (*n* = 3) and Dunnett's multiple comparison test was conducted for a.u. values of the capsule mutants relative to SGH10 wild type at 60 min. * denotes *p* < .05, ** denotes *p* < .001, while n.s. denotes not significant [Colour figure can be viewed at wileyonlinelibrary.com]

We reasoned that if the cell envelope defects of the ∆*wza* and ∆*wzy* mutants are due to the sequestration of Und‐P or accumulation of toxic capsule intermediates, inactivating *wcaJ*, the first step in capsule synthesis in these mutants would relieve and rescue the defect. Indeed, the ∆*wcaJ* and ∆*wzy* double mutant is as resistant to bile salt as the ∆*wcaJ* mutant, which is similar to the wild‐type bacteria (Figure 4a). Furthermore, when we complemented *wcaJ* into the double mutant, the strain showed increased sensitivity to bile salt (Figure 4b). Conversely, complementing *wzy* into the double mutant did not change the bile salt sensitivity of the strain compared to that of the double mutant. We also show that the ∆*wza* and ∆*wzy* mutants were more sensitive to perturbation with the detergent sodium dodecyl sulfate (SDS) compared to ∆*wcaJ*, ∆*wcaJ* ∆*wzy* mutants and the wild‐type bacteria (Figure 4c). This supports our hypothesis that cell envelope defects are likely a result of sequestration of Und‐P and its unavailability for other processes which support membrane integrity, or due to toxic build‐up of capsule intermediates downstream of *wcaJ*.

### 2.4 Capsule is not required for persistence in the gut

**Figure 5:**
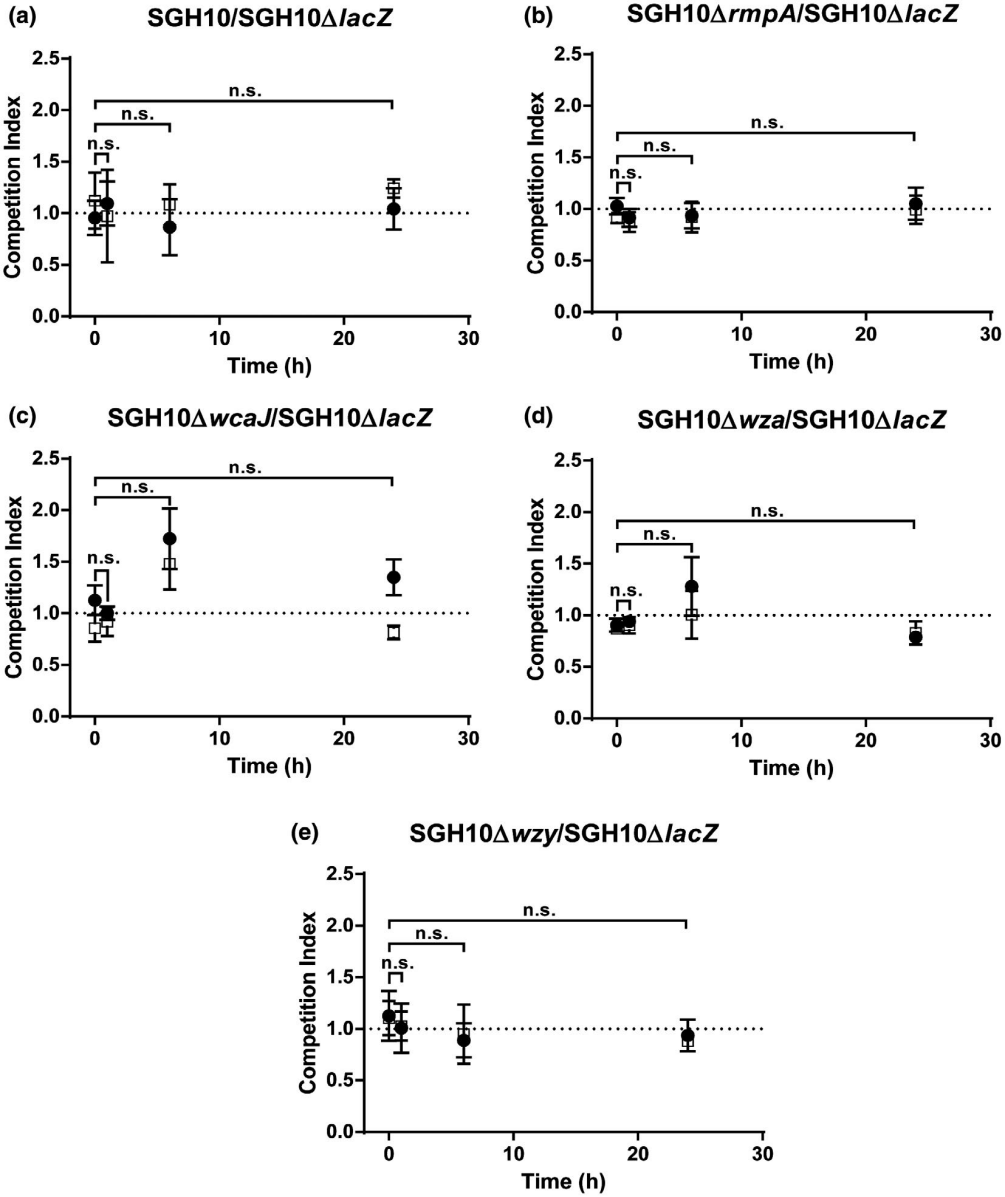
In vitro competition assays to determine relative fitness of mutants was performed between (a) SGH10 and SGH10Δ*lacZ*, (b) SGH10Δ*rmpA* and SGH10Δ*lacZ,* (c) SGH10Δ*wcaJ* and SGH10Δ*lacZ,* (d) SGH10Δ*wza* and SGH10Δ*lacZ* and (e) SGH10Δ*wzy* and SGH10Δ*lacZ.* Bacteria were inoculated into LB (circles) and 10 g/L pig mucin in PBS (open squares) at a starting ratio of 1:1, and mutant of interest and SGH10Δ*lacZ* were quantified at appropriate intervals to calculate competitive index (CI). Mean ± *SD* are plotted (*n* = 3) and * denotes *p* < .05, while n.s. denotes not significant. A CI value of 1 is taken to denote that the strains are equally fit. A CI value of <1 indicates that the mutant of interest is less fit than SGH10Δ*lacZ*, while a CI value of >1 indicates that the mutant is more fit than SGH10Δ*lacZ*. There is no statistically significant differences in all comparisons

While capsule production may be beneficial for hvKP to survive in the host, its synthesis may pose a significant metabolic burden (Lin et al., 2013). To investigate the role of capsule in hvKP virulence, we compared the fitness of each mutant relative to SGH10∆*lacZ* in the nutrient‐rich LB media or in PBS supplemented with 10 g/L pig mucin that more closely mimics the condition in the mammalian gut. SGH10∆*lacZ* was used in place of SGH10 during competitive assays to facilitate blue‐white colony selection. SGH10∆*lacZ* was first shown to be a viable surrogate for wild type in this in vitro competition assay, as the competitive index of wild type to SGH10∆*lacZ* was close to 1 throughout the assay (Figure 5a). Using the same in vitro assay, we show that all capsule mutants were as fit as SGH10∆*lacZ* with the competitive index close to 1 and grew at a similar rate as the SGH10∆*lacZ* control (Figure 5b–e).

**Figure 6:**
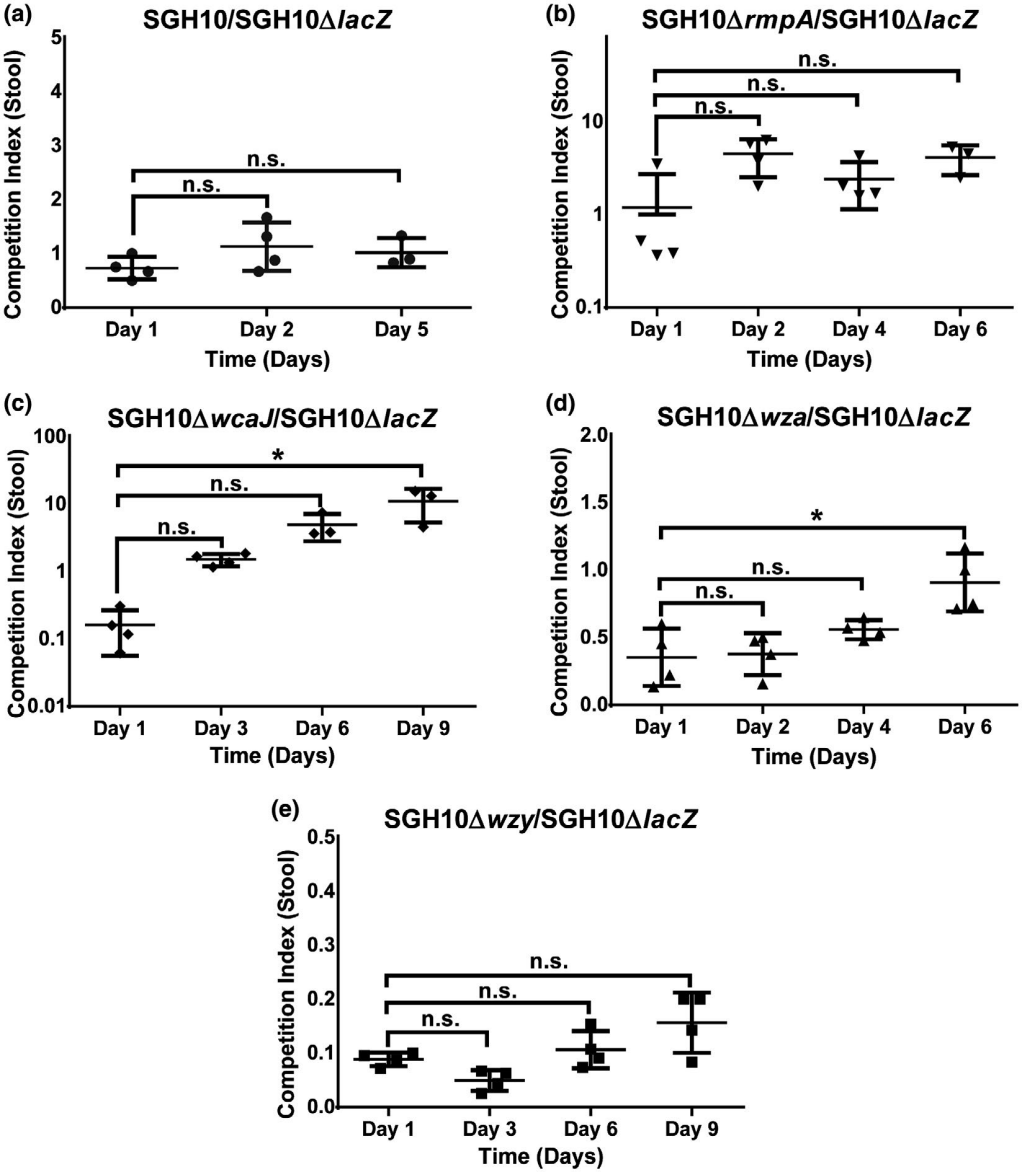
An in vivo competition assay to determine relative fitness of mutants in the mouse gut. (a) SGH10 and SGH10Δ*lacZ*, (b) SGH10Δ*rmpA* and SGH10Δ*lacZ,* (c) SGH10Δ*wcaJ* and SGH10Δ*lacZ*, (d) SGH10Δ*wza* and SGH10Δ*lacZ* and (e) SGH10Δ*wzy* and SGH10Δ*lacZ.* Bacteria were inoculated into wild‐type C57/BL6J mice via oral gavage at a starting ratio of 1:1 in order to determine if the mutant of interest is less fit than, or outcompetes SGH10Δ*lacZ.* At appropriate intervals, the mutant of interest and SGH10Δ*lacZ* were quantified in mouse stool to determine the CI of these strains in the gut. Mean ± Standard Error of the Mean (*SEM*) were plotted and * denotes *p* < .05, while n.s. denotes not significant. A CI value of 1 is taken to denote that the strains are equally fit. A CI value of <1 indicates that the mutant of interest is less fit than SGH10Δ*lacZ*, while a CI value of >1 indicates that the mutant is more fit than SGH10Δ*lacZ*

Next, we compared the ability of the capsule mutants and SGH10∆*lacZ* to persist in the murine gut by conducting an in vivo competition assay. In this competition assay, ampicillin pretreated mice were gavaged with the mutant of interest and SGH10∆*lacZ* in a ratio of 1:1, and bacteria were plated from stools over the course of the assay. SGH10 was as fit as SGH10∆*lacZ* in the mouse gut as the competitive index was about 1, which indicates that SGH10∆*lacZ* is a valid surrogate control for this assay (Figure 6a). SGH10∆*rmpA* outcompeted SGH10∆*lacZ* in the mouse gut; the initial competitive index of SGH10∆*rmpA*: SGH10∆*lacZ* was 1, but progressively increased to ≈4 over 6 days (Figure 6b).

SGH10∆*wcaJ* was initially less fit than wild type as the competitive index of SGH10∆*wcaJ*: SGH10∆*lacZ* was 0.1 on day 1. SGH10∆*wcaJ* eventually outcompeted SGH10∆*lacZ;* the competitive index was 10 after 9 days, which indicates that capsule is not required to maintain colonisation in the gut, although it aids in initial establishment of colonisation (Figure 6c). SGH10∆*wza* and SGH10∆*wzy* were less fit than SGH10∆*lacZ*. Throughout the in vivo competition assay, the competitive indices of SGH10∆*wza* and SGH10∆*wzy* to SGH10∆*lacZ* were less than 1 (Figure 6d,e). This effect is likely due to SGH10∆*wza* and SGH10∆*wzy* harbouring cell envelope defects that result in bile sensitivity, rather than the loss of capsule production or export.

### 2.5 Role of capsule and *rmpA* in systemic infection

**Figure 7:**
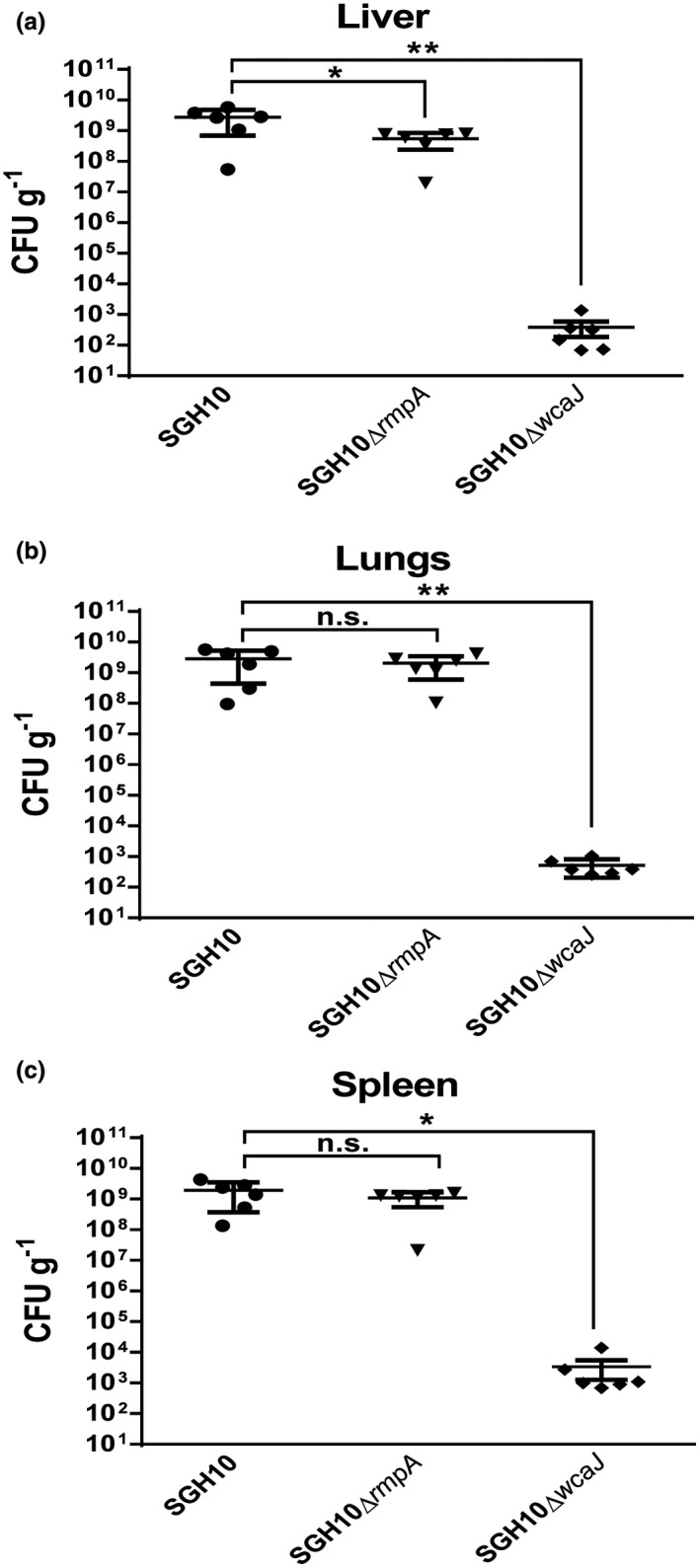
Intraperitoneal infection of C57BL/6J mice with SGH10, SGH10Δ*rmpA* and SGH10Δ*wcaJ*. (a) Bacterial loads in the liver, (b) lungs and (c) spleen were measured by plating at 30 hr post infection. Mean ± *SEM* are plotted and * denotes *p* < .05, while ** denotes *p* < .001

In many pathogenic bacteria, capsular polysaccharide is important for survival during systemic infection (Geno et al., 2015; Lawlor, Hsu, Rick, & Miller, 2005; Lee et al., 1987; Sahin et al., 2017). In hvKP, this has been shown using ∆*wzy* mutants (Fang et al., 2004, 2010; Wu et al., 2008)**.** Since this mutant confers additional cell envelope defects, we therefore revisited the effect of capsular polysaccharide on systemic dissemination and survival in the murine model with the ∆*wcaJ* mutant. C57/BL6J mice were infected with wild‐type, SGH10∆*rmpA* and SGH10∆*wcaJ* bacteria via intraperitoneal injection. After 30 hr, the mice were sacrificed and the lungs, liver and spleen were harvested, homogenised and plated to enumerate the number of hvKP in each organ. Our results show that SGH10∆*rmpA* could disseminate in the bloodstream and was detected at comparable organ loads with SGH10 WT except in the liver (Figure 7a–c). SGH10∆*wcaJ* organ loads were significantly less than wild type, indicating that this strain is more susceptible to clearance by the immune system during systemic infection (Figure 7a–c). All wild type‐infected mice were hunched and exhibited limited mobility and had to be euthanised according to our disease scoring system, whereas the capsule‐null mutant‐infected mice were healthy at the end of the experiment. The ∆*rmpA* mutant‐infected mice had an intermediate phenotype, with only two out of six mice appearing to be hunched and with limited mobility. Together, the results confirm the critical role of the K1 capsule in hvKP pathogenesis.

## 3 DISCUSSION

*K. pneumoniae* is proposed to be of “critical priority” for research and development of new antibiotics on the list of the World Health Organization priority pathogens (Tacconelli et al., 2018). Among various strains and isolates of *K. pneumoniae*, hvKP is particularly dangerous because it has the potential to acquire multi‐drug resistance and is capable of causing serious diseases like liver abscess, pneumonia and sepsis in relatively healthy individuals (Araújo et al., 2018; Chew, Lin, Teo, & Holt, 2017; Lee, Lee, Park, & Jeon, 2017; Roulston et al., 2018; Shon et al., 2013; Yao et al., 2015). The capsule of hvKP is a key virulence factor that aids dissemination to the host bloodstream and systemic infection (Alvarez et al., 2000; Cortés et al., 2002; Fang et al., 2004). Both spontaneous and specific capsule mutants for various capsule serotypes and mouse infection models have been used to study the role of *K. pneumoniae* capsule (Supporting Information Table S1). Different capsule mutants could give rise to divergent phenotypes due to different infection models as well as the properties of the mutants, given that capsule synthesis and export is a multi‐gene and complicated assembly line. We decided to examine the effect of using different isogenic capsule‐null mutants, particularly the commonly employed *wzy* and *wza* deletion mutants, in comparison with the *wcaJ* mutant, on bacterial pathogenesis, particularly in gut colonisation, as this has not been well established.

The thick capsule of hvKP could potentially prevent hvKP from adhering to the gut mucosa, causing it to be easily dislodged by peristalsis. While one study suggested that *K. pneumoniae* capsule reduced adhesion to ileocecal and bladder cells (Sahly et al., 2000), others have shown that *K. pneumoniae* capsule could enhance association to lung epithelial cells and intestinal epithelial cell lines (Clements et al. 2008; Favre‐Bonte et al., 1999). We show that the thick capsule of hvKP impedes its adhesion to colonic cell line Caco‐2 (Figure 2b). However, as hvKP grows at a relatively fast rate, clearance of weakly adhering bacteria through peristalsis may not significantly reduce colonisation of the gut. Struve and Krogfelt previously used noncapsulated variants of *K. pneumoniae* to show that capsule is nonessential for gastrointestinal colonisation of streptomycin‐treated mice (Struve & Krogfelt, 2003).

To definitively answer the question of whether the capsule is essential for gut colonisation, we constructed three isogenic capsule‐null mutants SGH10∆*wcaJ*, SGH10*∆wza* and SGH10*∆wzy*, as well as the capsule regulator mutant SGH10*∆rmpA*. By comparing these mutants and their phenotypes in different infection models, we sought to identify the changes that are solely attributed to the loss of capsule. Deletion of *wza* will lead to the inability to export capsular polysaccharide (Wei, Yuminaga, Shi, & Jian, 2018), while ∆*wzy* deletion will stop polymerisation of the capsule precursor (Ho et al., 2011). Both the ∆*wza* and ∆*wzy* mutants will accumulate capsule intermediates. However, in SGH10∆*wcaJ*, the capsule intermediates are not synthesised, and hence cells are less likely to exhibit membrane stress. Indeed, *SEM* showed that ∆*wza* and ∆*wzy* mutants exhibited aberrant cell shapes. These alterations are absent in the wild‐type as well as the ∆*wcaJ* mutant, suggesting inhibition of intermediate steps in capsule synthesis will result in cell envelope defects. Consistent with this observation, ∆*wza* and ∆*wzy* mutants are sensitive to bile salts, whereas the ∆*wcaJ* mutant is not.

We hypothesise that either the accumulation of dead‐end capsular polysaccharide precursors or sequestration of Und‐P caused the envelope defect in hvKP. In *E. coli*, the enterobacterial common antigen (ECA), O‐antigen and peptidoglycan synthesis pathways all compete for a single pool of Und‐P (Hartley & Imperiali, 2012; Hug & Feldman, 2011; Valvano, 2008). Genetic inactivation of factors required for intermediate steps of the ECA pathway induces morphological defects and swelling. This is due to the build‐up of ECA lipid II that sequesters the pool of Und‐P for peptidoglycan synthesis (Jorgenson & Young, 2016) because overexpression of Und‐P synthase UppS largely rescued the phenotype. Similarly, *E. coli* spheroplasts can resynthesise an intact cell wall unless colanic acid biosynthesis is interrupted by mutations downstream of the initial glycosyltransferase *wcaJ,* leading to spheroplast lysis (Ranjit & Young, 2016). In this case, it is the accumulation of colanic acid intermediates that inhibits spheroplast recovery because an increased level of UppS did not prevent lysis but deletion of *wcaJ* did. In *K. pneumoniae*, we found that the aberrant cell envelope in *wzy* and *wza* mutants did not change the growth rates, which indicates hvKP somehow can tolerate a lower level of Und‐P or there is an unidentified feedback loop that stops capsule production if the level of Und‐P becomes dangerously low. Our data with the double mutant of *wzy* and *wcaJ*, as well as complementation of the double mutant with either *wzy* or *wcaJ* show that the cell envelope defects are due to the accumulation of capsule intermediates. Whether the defects are due to toxic capsule intermediates, or to the sequestration and therefore depletion of Und‐P for peptidoglycan synthesis is unclear. Interestingly, the ∆*wza* mutant showed more resistance to bile salt and greater fitness in the gut compared to the ∆*wzy* mutant. If sequestration of Und‐P is the sole reason for cell envelope defects, these two mutants should have the same phenotype. Therefore, it is possible that the capsule intermediates in the ∆*wzy* mutant are contributing to cell wall defects. Together, our results show that ∆*wza* and ∆*wzy* deletion mutants should not be used to investigate the role of capsule in virulence due to secondary effects on cell envelope stability.

In this study, we clearly observed differences between the ∆*wcaJ* and the ∆*wza* or ∆*wzy* mutants in the gut. Strains SGH10∆*wza* and SGH10∆*wzy* have a competitive index <1 throughout the in vivo competition assay, demonstrating their poor ability to colonise and persist in the gut. On the other hand, SGH10∆*wcaJ* showed an initial disadvantage compared to the wild‐type hvKP, but this handicap was quickly rectified and eventually the unencapsulated mutant outcompeted the wild‐type bacteria in the gut. This outgrowth of the unencapsulated mutant at later points could be due to the fitness cost associated with the synthesis of the capsule. In contrast, the intraperitoneal infection model showed that the ∆*wcaJ* mutant had a drastic reduction in virulence and was readily cleared by the host immune system. The data agree with the ∆*wcaJ* mutant in a *K. pneumoniae* K7 strain, where it showed a decrease in virulence in the murine model of intranasal infection (Liu, Han, & Gu, 2019).

The hypermucoid nature of hvKP is contributed by *rmpA* which is typically encoded on the large virulence plasmid in hvKP, although it can also be found on the chromosome (Cheng et al., 2010; Hsu et al., 2011; Struve et al., 2015). RmpA regulates the expression of capsule genes by interacting with the phosphorelay response regulator RcsB (Hsu et al., 2011; Nassif, Fournier, Arondel, & Sansonetti, 1989). Plasmid‐encoded *rmpA* has also been shown to increase capsule mucoviscosity by increasing the expression of colanic acid in *Escherichia coli* (Nassif, Honore, Vasselon, Cole, & Sansonetti, 1989). As such, it has been postulated that this gene increases the expression of group I capsule in *K. pneumoniae* since the expression of colanic acid and group I capsule are mutually exclusive and the same initial *rcsB* binding site is present (Cheng et al., 2010; Hsu et al., 2011; Lai, Peng, & Chang, 2003). Previous work with the serotype K2 strain CG43 and the K1 strain NTUH‐K2044 concluded that RmpA is important for systemic infection in the former but not the latter (Cheng et al., 2010; Hsu et al., 2011; Nassif, Fournier, et al., 1989). The intraperitoneal infection by NTUH‐K2044 was performed in a competitive assay with wild‐type bacteria, showing that a competitive index of >5 when bacteria were plated from the liver at 24 hr post infection. This competitive advantage of the ∆*rmpA* mutant is similar to our observations with the SGH10∆*rmpA* mutant in competition with wild‐type bacteria during gut colonisation. This indicates that the ∆*rmpA* mutant could have a growth advantage not only in the gut but also in the liver. However, in our intraperitoneal infection of 30 hr, we found that the bacterial loads in the liver were lower in the ∆*rmpA* mutant compared to the wild type‐infected mice, although the bacterial loads were similar in other organs. This discrepancy could be due to strain differences because NTUH2044 has a functional chromosomal copy of ∆*rmpA* as well as the plasmid copy. The competitive advantage of ∆*rmpA* in the liver could be due to initial bystander protection mediated by the wild type. Our intraperitoneal infection data on the ∆*rmpA* mutant is in line with a previous study using intraperitoneal infection (Nassif, Fournier, et al., 1989). Walker et al. (2019) showed that the ∆*rmpA* mutant was less virulent than wild type during intranasal infection and bacterial loads in the lungs were significantly reduced. In the intraperitoneal route of infection, we did not see a decrease in lung bacterial loads with the *∆rmpA* mutant, but overall the disease was less severe in these mice compared with those infected wild‐type SGH10. This suggests that *rmpA* plays a smaller role in the pathogenesis of SGH10.

Bile salts are produced in the liver and subsequently transported to the intestinal tract (Pellicoro & Faber, 2007; Russell, 2009). Given hvKP’s propensity to colonise the gut and cause liver disease, the ability to resist bile stress is likely crucial. For most *Enterobacteriaceae* including *Klebsiella*, resistance to bile stress requires an intact outer membrane (Merritt & Donaldson, 2009; Urdaneta & Casadesús, 2017; Vianney, Lazzaroni, & Germon, 1999), as no bile salt hydrolases have been reported thus far in *Klebsiella*. SGH10∆*wcaJ* is resistant to bile stress, which indicates that the capsule is not required for resistance to bile stress. Both ∆*rmpA* and ∆*wcaJ* mutants exhibited a competitive index of <1 in less than one day post inoculation. This suggests that the lower ability to initiate colonisation without a capsule or with a nonhypermucoid capsule is not related to bile salt stress but could be due to other physical or chemical factors initially encountered in the gut environment. However, the mutants were able to gain the upper hand quickly and outcompeted the wild‐type bacteria, indicating that capsule is not required for colonisation persistence, and in fact might represent a metabolic burden in the gut environment. Given that RmpA is able to regulate many other genes besides capsule, these other genes may additionally impede colonisation persistence (Hsu et al., 2011; Walker et al., 2019).

In conclusion, we advise caution in the interpretations of phenotypic results of *K. pneumoniae* ∆*wza* and ∆*wzy* mutants that are attributable to loss of capsule as these mutants have additional cell envelope defects due to accumulation of capsule intermediates that was not previously appreciated. Our work establishes that capsule is not essential for the persistence of K1 hvKP in the gut although it is important for systemic infection.

## 4 EXPERIMENTAL PROCEDURES

### 4.1 Maintenance of bacterial cultures

SGH10 was originally isolated from a liver abscess patient and is a reference strain for *K. pneumoniae* CG23 sublineage 1 deposited at the National Collection of Type Cultures at the United Kingdom (NCTC 14052) (Lam et al., 2018; Lee et al., 2016).

SGH10Δ*rmpA*, SGH10Δ*wza*, SGH10Δ*wzy*, SGH10Δ*wcaJ* and SGH10Δ*lacZ* are clean deletion mutants generated in this study. All bacterial strains were cultured in Lysogeny broth (LB) and on LB agar plates and 30 μg/ml trimethoprim (Sigma‐Aldrich) was used to maintain the pUCP28T plasmid (p28T) (West et al., 1994).

### 4.2 Density‐based separation of bacteria

Two millilitres of 50%, 35% and 15% Percoll in 1× PBS (Sigma‐Aldrich) were aliquoted into a 15 ml falcon tube from highest to lowest density. The strain of interest measuring 1.8 × 10^11^ CFU in 600 μl of 1× PBS was added dropwise to the tube. The tubes were then centrifuged at 3,000 × g for 30 min and imaged against a black background.

### 4.3 Low‐speed centrifugation of bacteria

Six millilitres of 10^9^ CFU/ml of the strain of interest in LB were prepared in 15 ml falcon tubes. The tubes were then centrifuged at 2,000 × g for 10 min and imaged against a black background. Optical density 600 nm (O.D. 600 nm) readings of the supernatant were then taken using a BioMate^TM^ spectrophotometer (Thermo Scientific).

### 4.4 FITC–dextran exclusion assays

Overnight cultures of the strain of interest were grown in LB and resuspended in 1× PBS at 10^10^ CFU/ml. One microlitre of bacterial culture and 1 μl of 10 mg/ml Fluorescein isothiocyanate (FITC)–dextran in 1× PBS (Sigma Aldrich) were mixed and spotted onto a glass slide and covered with a glass coverslip. Bacteria were imaged for differential contrast (Brightfield) and fluorescence using an Olympus IX81 inverted microscope equipped with an Olympus DP26 camera for image acquisition with a 100X oil immersion objective lens. Images were formatted and analysed using ImageJ. Bacterial capsule appears as a grey shadow against the green background of FITC–dextran in the merged image.

### 4.5 Mammalian cell culture

Mammalian cells were maintained in a CO_2_ incubator (Themo Scientific) at 37°C with 5% CO_2_. RAW 264.7 cells (ATCC) were grown in Dulbecco's modified Eagle's medium (DMEM) (Life Technologies) supplemented with 10% fetal bovine serum (FBS) (Singlab) and 1X penicillin–streptomycin (Singlab). Caco‐2 cells (ATCC) were grown in DMEM supplemented with 20% FBS, 1X penicillin–streptomycin and 1X nonessential amino acids (Sigma‐Aldrich).

### 4.6 Serum resistance assay

Twenty‐five microlitres of 2.5 × 10^5^ CFU of *K. pneumoniae* in 1× PBS (Thermo Scientific) were inoculated into wells of a 96‐well plate containing 75 μl of 100% pooled normal human serum (Sigma‐Aldrich). Control serum was inactivated by heating at 56°C for 30 min prior to incubation of bacteria. The plate was then incubated at 37°C. At 0, 1, 2 and 3 hr post inoculation, appropriate dilutions were plated on LB agar to enumerate surviving bacterial colony‐forming units (CFU).

### 4.7 Bacterial phagocytosis and adhesion assays

For phagocytosis assays, RAW 264.7 cells were seeded at 0.125 million cells in a 24‐well plate and allowed to attach overnight. Cells were then washed with 1× PBS and 0.5 ml fresh DMEM + 10% FBS was added. *K. pneumoniae* was added to achieve a Multiplicity of Infection (MOI) of 1:1 and the plate was centrifuged at 250 × g for 5 min. After 1 hr, kanamycin was added to give a final concentration of 500 μg/ml kanamycin. At 2 hr post infection, cells were lysed with 0.2% Triton‐X100 in 1× PBS (Sigma‐Aldrich) and appropriate dilutions were plated on LB agar to enumerate bacterial CFU.

For cell adhesion assays, 0.25 million Caco‐2 cells/well were seeded in a 24‐well plate and allowed to attach overnight. Cells were then washed with 1× PBS and fresh DMEM + 10% FBS was added. *K. pneumoniae* was added to the well to give an MOI of 10:1 and the plate was centrifuged at 250 × g for 5 min. After a 30 min incubation, cells were washed twice with 1× PBS and lysed with 0.2% Triton‐X100 in 1× PBS (Sigma‐Aldrich). Appropriate dilutions were plated on LB agar to enumerate bacterial CFU.

### 4.8 In vitro bacterial competition assays

SGH10Δ*lacZ,* 10^3^ CFU, and 10^3^ CFU of the mutant of interest (initial ratio of 1:1) were inoculated in 5 ml of 10 g/L mucin in 1× PBS or LB in a 50 ml falcon tube and incubated at 37°C with shaking. At 0, 1, 6 and 24 hr, appropriate dilutions of the bacterial culture were plated on LB agar spread with 40 μl of 1 mM Isopropyl β‐D‐1‐thiogalactopyranoside (IPTG) and 40 μl of 20 mg/ml 5‐bromo‐4‐chloro‐3‐indolyl‐D‐galactopyranoside (X‐gal). SGH10Δ*lacZ* is unable to degrade X‐gal and hence produces white colonies, while the competing strain appears as blue colonies. The ratio of competing strain: SGH10Δ*lacZ* was then calculated to give the Competitive Index (CI) value. A CI value of <1 indicates that the mutant of interest is less fit than wild type, while a CI value of >1 indicates that the mutant of interest outcompetes the wild type.

### 4.9 Bacterial colony‐forming unit assay

LB agar plates supplemented with appropriate concentrations of sodium deoxycholate (DOC), sodium cholate (CHO) and LB agar adjusted to pH4 with hydrochloric acid were prepared by boiling agar with the appropriate detergent in a water bath. Overnight cultures of bacteria were diluted in 1× PBS and 5 μl of bacterial suspension were spotted on each plate. Tenfold dilutions were plated in each drop starting with 10^5^ CFU–10 CFU, for a minimum of eight plates per condition. The plates were allowed to dry before overnight incubation in a 37°C incubator and imaged using a BioRad ChemiDoc^TM^ MP Imaging System.

### 4.10 Propidium iodide accumulation assays

Bacteria were grown on LB agar plates to mid‐log phase (4–5 hr) and washed off with 1× PBS. Bacterial concentration was adjusted to O.D. 600 nm = 0.5. RNase A (final concentration of 4 μg/ml) and propidium iodide (final concentration of 2 μg/ml) were added to bacterial suspensions. The suspensions were aliquoted in Corning Clear Bottom Black Side plate at 90 μl per well in triplicates. Sodium dodecyl sulfate (SDS) was added to each well to achieve a final concentration of 0.5%. The plate was read and recorded every minute for 1 hr (excitation 305 nm, emission 617 nm) and fluorescence intensity was captured in arbitrary units (a.u.).

### 4.11 Construction of gene deletion mutants in *K. pneumoniae*

Clean deletion mutants were generated in *rmpA* (SGH10_RS27810), *wza* (SGH10_RS08555), *wzy* (SGH10_RS08575), *wcaJ* (SGH10_RS08610) and *lacZ* (SGH10_RS14285). For generation of gene deletion mutants in *K. pneumoniae*, a conditional suicide vector was constructed by replacing the pMB1 replication origin in pK18mobsacB (Kvitko & Collmer, 2011) with the R6K replication origin from EZ‐Tn5™ <R6Kγ ori/KAN‐2 > Transposon (Epicentre) to generate pR6KmobsacB. To delete a gene in *K. pneumoniae*, ∼1000‐bp fragments upstream and downstream from the gene of interest were PCR amplified from genomic DNA templates with Q5 High‐Fidelity DNA Polymerase (New England Biolabs) and then assembled in the pR6KmobsacB vector with NEBuilder® HiFi DNA Assembly Master Mix (New England Biolabs). A list of all primers used and their sequences can be found in Table 1.

**Table 1:** List of the primers used for cloning and mutant generation

Primer name	Sequence
wcaJ Up‐for	ACATGATTACGAATTCTGCCTCTGGTAAAGGAGTGC
wcaJ Up‐rev	GCATCTTAGAAGATTTCCTTAAAATATTGAACCTT
wcaJ Dn‐for	AAATCTTCTAAGATGCTGCTTAAGATAAGCATTGT
wcaJ Dn‐rev	TTGCATGCCTGCAGAGCTCTGGCTGGTCCACTTA
wza Up‐for	ACATGATTACGAATTCTCTCCGCCAGCCAAATGAAT
wza Up‐rev	ATTTAAACATAATGTCACATCATTAGTAAACCAAGATTGC
wza Dn‐for	TGTGACATTATGTTTAAATCAGTTTTAGTTGTTTGTATTGG
wza Dn‐rev	CTTGCATGCCTGCAGTCGCCGGCATCATCAAGATT
wzy Up‐for	ACATGATTACGAATTCTGCCCTGATGCCTTTAGTTT
wzy Up‐rev	CTTTTTGCATCAAATAAAATCACACATTGCATCATTACC
wzy Dn‐for	TTTATTTGATGCAAAAAGATTTAAAATAATAGAGGAAGAACAAT
wzy Dn‐rev	CTTGCATGCCTGCAGCAACCCGGTCTGTAATGCCT
lacZ Up‐for	TATGACATGATTACGAATTCGCCATCTGATCGTTTGCCAC
lacZ Up‐rev	CCACGGATTACATATTAAACCCCGGTAAGT
lacZ Dn‐for	GTTTAATATGTAATCCGTGGGGGCGACAGC
lacZ Dn‐rev	CCAAGCTTGCATGCCTGCAGCCGAGAATACGCACCGACAT
rmpA Up‐for	CTTGCATGCCTGCAGCTTTAGTTAAGGCGGCCTTCG
rmpA Up‐rev	CTAGAGGATCCCCGGGTACCCATAGAAACAGTAACTTTGATCCATCAATATTCATCC
rmpA Dn‐for	CCCGGGGATCCTCTAGAGTCTAGGTAAAAAAGGGGAGGGGATG
rmpA Dn‐rev	ACATGATTACGAATTCTGAGCCAAATGTATGCCAAGG
wcaJ‐for	ACATGATTACGAATTCCGCATCACTTGATATGAATAATGTACT
wcaJ_rev	TTGCATGCCTGCAGGGTTGAAAACGGAGACGGTA
wza‐for	ACATGATTACGAATTCCCAGCGCAGGGATAGAAATA
wza‐rev	TTGCATGCCTGCAGCCGATCTTGAAATCTTTCAGTCTT
wzy‐for	ACATGATTACGAATTCAACTTGAACGAGCAATTCAATC
wzy‐rev	TTGCATGCCTGCAGATGGCCATTTGCGTTAGTACA

Plasmids were introduced into *K. pneumoniae* via conjugation from *E. coli* donor strain S17‐1λpir. Single crossover transconjugants were selected on medium containing kanamycin (50 μg/ml) and donor *E. coli* was removed by carbenicillin (100 μg/ml) in medium. Kanamycin‐resistant, single crossovers were passaged in LB medium lacking sodium chloride plus 20% sucrose to counter select the *sacB* gene on the pR6KmobsacB backbone. Kanamycin‐sensitive, double crossovers were screened for successful deletion of the gene of interest by polymerase chain reaction. To create Δ*wcaJ* Δ*wzy* double mutant, the *wzy* deletion construction in pR6K plasmid was introduced into Δ*wcaJ* single mutant. Sucrose‐counterselection and double‐crossover screening were carried out as above.

### 4.12 Scanning electron microscopy

Overnight cultures of bacteria were pelleted at 2,000 × g for 7 min and washed with 1× PBS (pH 7.3). The bacteria were then fixed in 2.5% glutaraldehyde (Sigma‐Aldrich) in 1× PBS (pH 7.3) for 2 hr at 4°C. Subsequently, bacteria were washed with 1× PBS (pH 7.3) twice with same centrifugal conditions at 2,000 × g, 7 min. Samples were stored at 4°C. The bacteria samples were prepared by spinning at 2,000 × g for 7 min, fixed onto sample stub for gold sputtering and imaged using scanning electron microscopy. Microscopy imaging was done with the assistance of the Electron Microscopy Unit of Yong Loo Lin School of Medicine, National University of Singapore.

### 4.13 Animal work

For all experiments, female C57 BL/6 JAX® mice aged 7–8 weeks were purchased from InVivos. For the in vivo bacterial competition assays, mice were orally gavaged with 2.5 mg of ampicillin sodium salt (Sigma‐Aldrich) in 100 μl 1× PBS daily for 5 days. On the subsequent day, 2.5 × 10^6^ CFU of SGH10Δ*lacZ* and 2.5 × 10^6^ CFU of the mutant of interest (a ratio of 1:1) was prepared in 100 μl 1× PBS and inoculated into mice via oral gavage. At suitable time points, stools were collected from each mouse. Serial dilutions of homogenised stool in 1× PBS were plated on *Klebsiella*‐selective agar (Sigma‐Aldrich) spread with 40 μl of 1 mM IPTG and 40 μl of 20 mg/ml X‐gal. SGH10Δ*lacZ* is unable to degrade X‐gal and hence produces white colonies, while the competing strain appears as blue colonies. The ratio of competing strain: SGH10Δ*lacZ* was then calculated to give the CI value.

For intraperitoneal infection of mice, 10^5^ CFU of each strain was prepared in 100 μl 1× PBS and inoculated into mice via intraperitoneal injection. At 30 hr post infection, mice were sacrificed and serial dilutions of homogenised liver, lungs and spleen in 1× PBS were plated on *Klebsiella*‐selective agar.

### 4.14 Ethics statement

The protocol and procedures employed for animal work in this study were ethically reviewed and approved by the NUS Institutional Animal Care and Use Committee (IACUC) in the animal facility at Comparative Medicine, National University of Singapore (NUS). The care and use of animals for research and teaching in NUS is bound by the Singapore Animals and Birds Act, Animals and Birds (Care and Use of Animals for Scientific Purposes) Rules 2004 and is carried out in accordance with the National Advisory Committee for Laboratory Animal Research (NACLAR) Guidelines. For this study, animals were used under Protocols R15‐135 and R18‐0252 as approved by the NUS IACUC.

### 4.15 Statistical methods

All graphical and numerical data were plotted using Graphpad Prism 6.0 (GraphPad Software, La Jolla California USA, www.graphpad.com). Student's *t*‐test was used to compare means and standard deviations (*SD*) and Dunnett's multiple comparison's test was used to compare means relative to wild‐type bacteria. A *p* value of < .05 is considered statistically significant and denoted as * and a *p* value < .001 is denoted as **.

## AUTHOR CONTRIBUTIONS

YHT and YHG conceptualised and designed the study. YHT performed all the experiments with the aid of YC for the generation of bacterial mutants and WHWC for animal work and *SEM*. YHT, LTS and YHG analysed data. YHT and YHG wrote the manuscript with contributions from the rest. All authors approved and vetted this manuscript.

## Data Availability

The data that support the findings of this study are available from the corresponding author upon reasonable request.
