# Supplementary material for: Cell envelope defects of different capsule‐null mutants in K1 hypervirulent Klebsiella pneumoniae can affect bacterial pathogenesis
Source: Mol Microbiol. 2020 Jan 20;113(5):889–905. doi: 10.1111/mmi.14447 (PMC7317392; doi:10.1111/mmi.14447)
Supplement: Supplementary file 1 [file MMI-113-889-s001.docx]

**Cell envelope defects of different capsule-null mutants in K1 hypervirulent *Klebsiella pneumoniae* can affect bacterial pathogenesis**

Running title: Capsule defects of hypervirulent *Klebsiella* affect gut colonisation

Supplementary Information.

**Yi Han Tan^1^, Yahua Chen^1^, Wilson HW Chu^1^, Lok-To Sham^2^ and Yunn-Hwen Gan^1^***

^1^ Department of Biochemistry, Yong Loo Lin School of Medicine, National University of Singapore, Singapore.

^2^ Department of Microbiology and Immunology, Yong Loo Lin School of Medicine, National University of Singapore, Singapore.

* Corresponding author

E-mail: bchganyh@nus.edu.sg

Supporting Information

Supplementary Figure 1. Capsule mutants grow in heat-treated serum. (A-D) Capsule mutants and their complemented counterparts were grown in heat-treated 75% human serum as a control to demonstrate that susceptibility to serum is due to complement sensitivity. Mean ± SD are plotted (n=3), while n.s. denotes not significant.

Supplementary Figure 2. FITC-dextran exclusion assay for capsule visualization. SGH10 and capsule mutants were visualised against a green background of FITC-dextran. Wildtype bacteria are surrounded by a grey halo of capsule while unencapsulated mutants appear as black cells against green background without a halo when both FITC and brightfield images are merged. The scale bar is 2 μm.

Supplementary Figure 3. Bacterial capsule is not required for resistance to pH stress. SGH10 and capsule mutants were plated on LB agar (pH4 and pH7). SGH10 capsule mutants were as fit as wildtype at pH4.

**Supplementary Table 1. The use of different mutants to study the role of capsule in Klebsiella pneumoniae virulence and pathogenesis in *in vivo* mouse models**

**Supplementary Figure 1.**


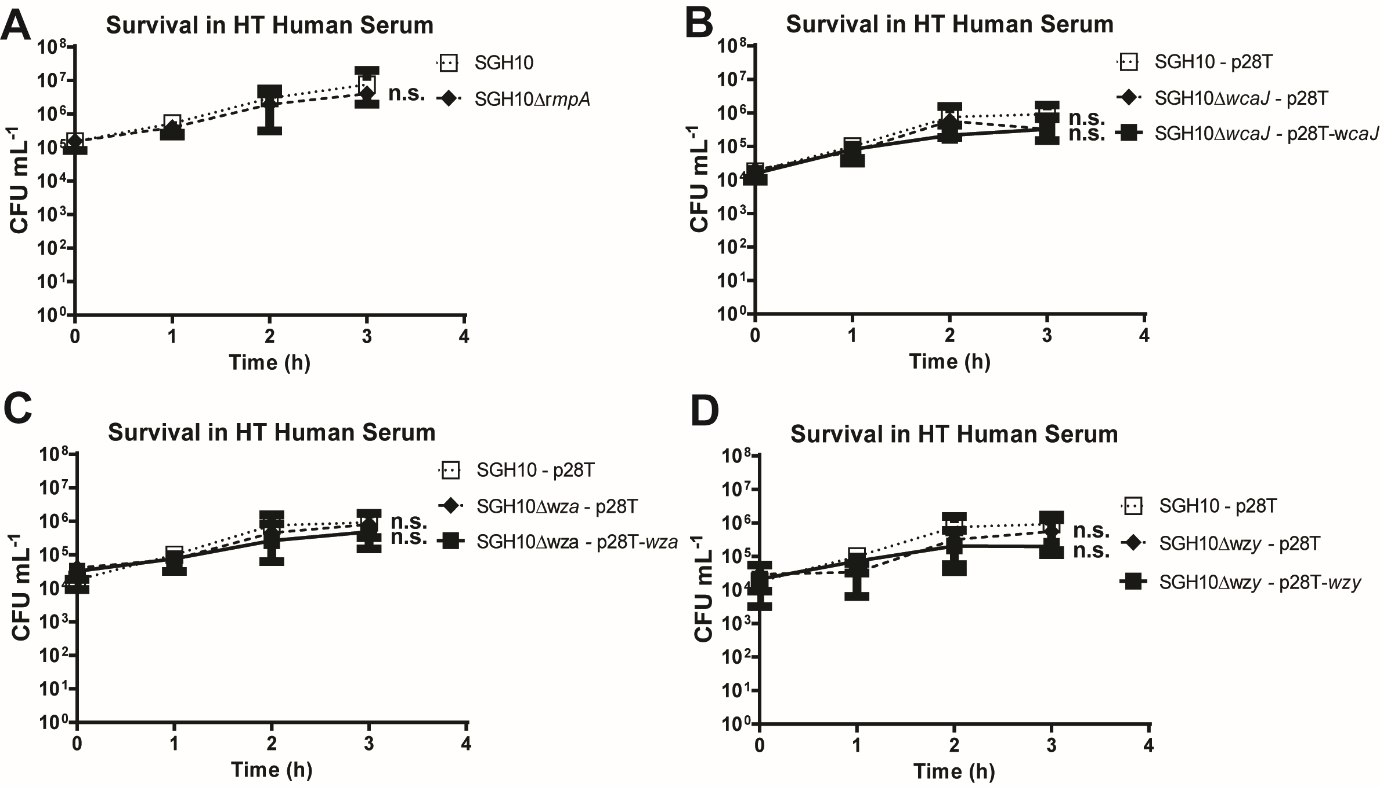


**Supplementary Figure 2.**


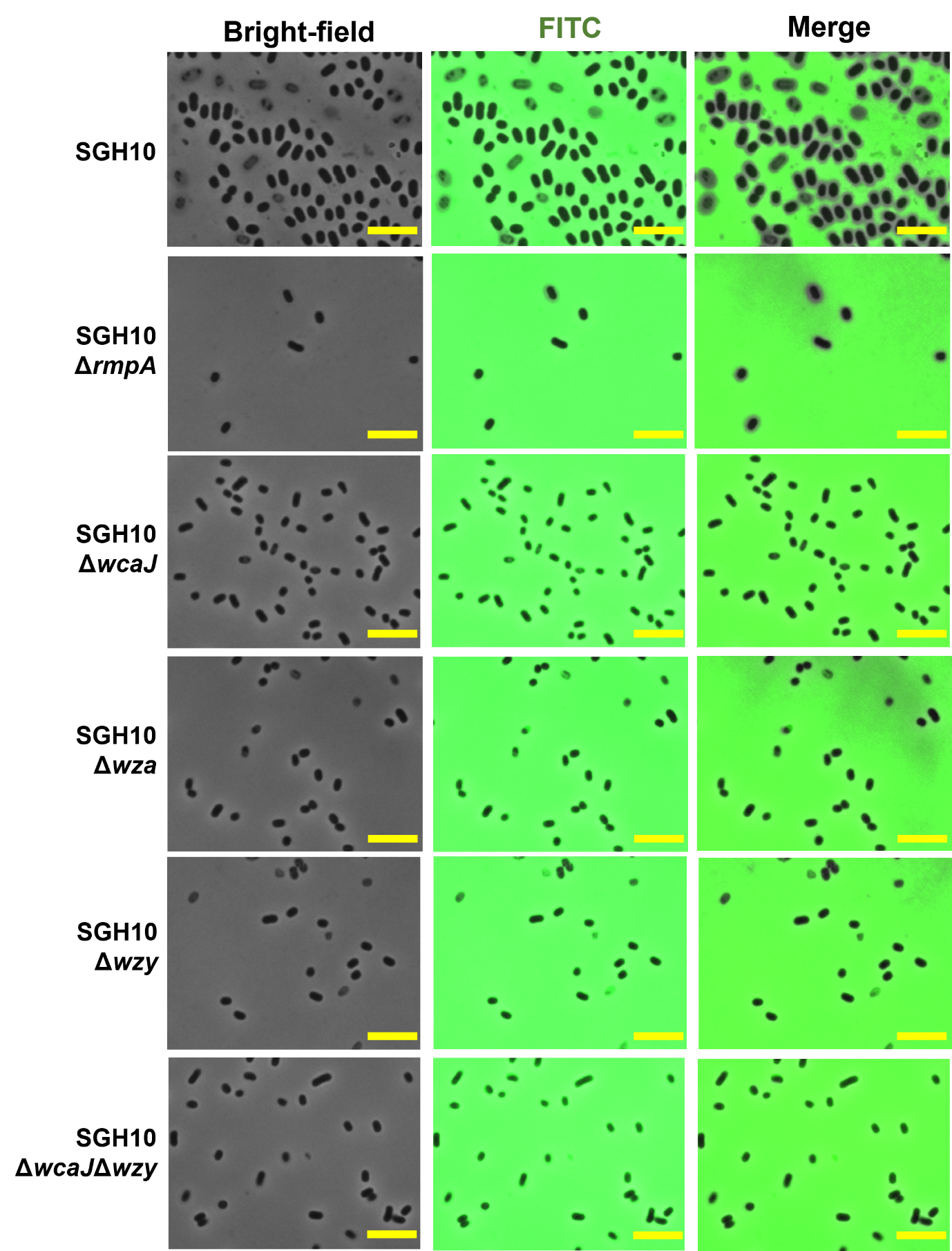


**Supplementary Figure 3.**


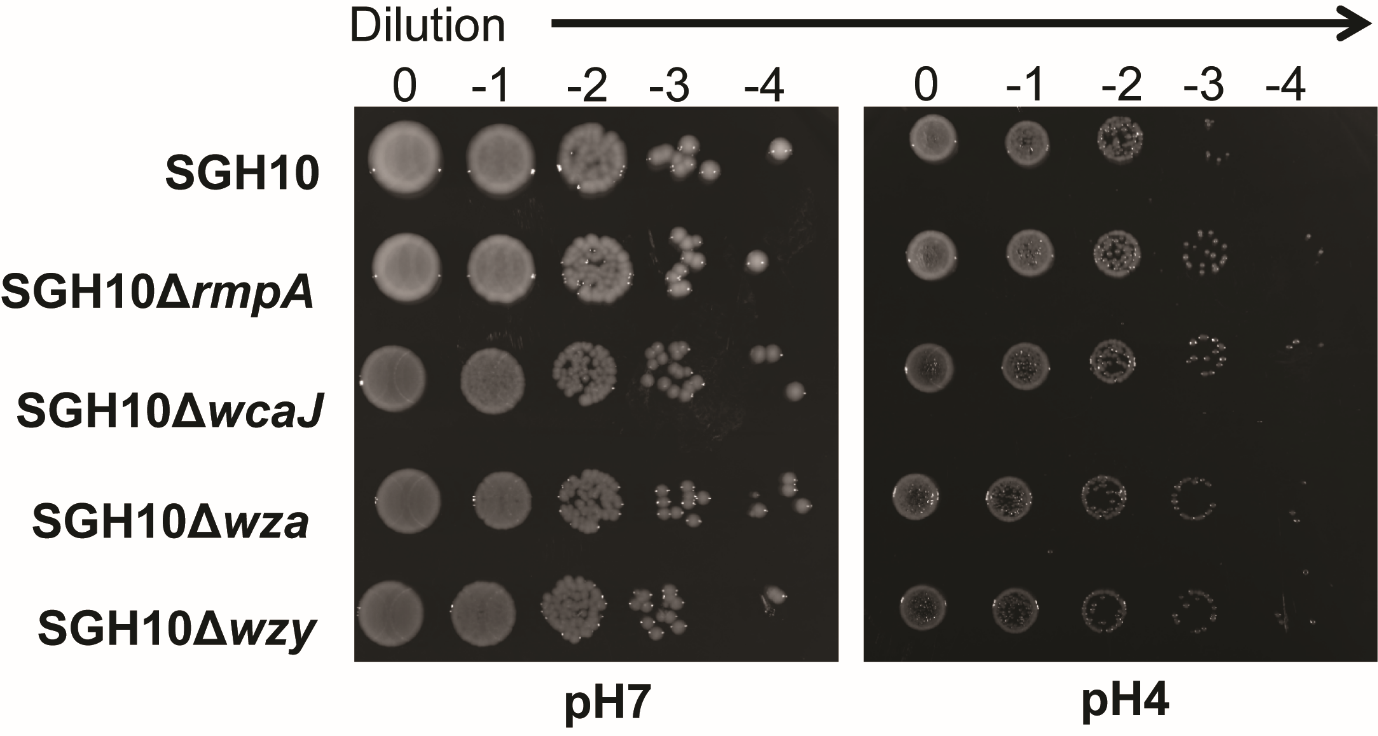


**Supplementary Table 1.** The use of different mutants to study the role of capsule in *Klebsiella pneumoniae* virulence and pathogenesis in *in vivo* mouse models

| ***K. pneumoniae* Strain** | **Capsule Type** | **Mutant** | **Route of infection** | **Organs** | **Findings** | **Study** |
| --- | --- | --- | --- | --- | --- | --- |
| C105  C3019 | K35  K2 | Non-capsulated variants | Intragastric inoculation  Bladder infection via catheter | Gut colonisation  Ascending urinary tract infection | No difference in gut colonisation in non-capsulated variants  Non-capsulated variants were attenuated | (Struve and Krogfelt 2003) |
| LM21 | K35 | Capsule genomic cluster was deleted | Streptomycin-treated mice were fed bacteria to induce colonisation | Gut colonisation | Capsule defective mutant was attenuated in gut colonisation | (Favre-Bonté *et al*. 1999) |
| LM21 | K35 | Capsule genomic cluster was deleted | Intragastric inoculation of ampicillin treated mice | Gut colonisation | Capsule defective mutant was attenuated in gut colonisation | (Maroncle *et a*l. 2002) |
| *rmpA*-negative and *wzy*-negative isolates (not isogenic mutants) | K1  K2  K62 | *rmpA*  *wzy* | Intraperitoneal injection | Systemic infection, liver | Attenuated | (Fung *et al*. 2011) |
| *rmpA*-negative and *wzy*-negative isolates (not isogenic mutants) | K1  K2  Non-K1/K2 | *rmpA*  *wzy* | Intraperitoneal injection | Systemic infection | *rmpA*-negative and *wzy*-negative strains are attenuated in virulence | (Yeh *et al*. 2007) |
| NVT-1001  NVT-20312 | K1  K20 | *galF*  *wzi*  *wza*  *wzb*  *wzc* | Intraperitoneal injection | Systemic infection | Attenuated 10-fold  Attenuated100 fold compared to WT, capsule likely retained  Attenuated 10^5^ fold  Attenuated 10^5^ fold  Attenuated 10^5^ fold | (Lin *et al*. 2017) |
| NTUH-2044 | K1 | *wzy* | Intraperitoneal injection | Systemic infection | Attenuated | (Ho *et al*. 2011) |
| STL43 | K1 | *wzy* | Intraperitoneal injection | Systemic infection, liver | Attenuated | (Yeh *et al*. 2016) |
| NTUH-2044 | K1 | *atf*  *ptf*  *glf*  *uge*  *gnd*  *wbaP*  *wcaI*  *wcaH*  *wcaG*  *wcfG* | Intraperitoneal injection | Systemic infection | Attenuated  Attenuated  No loss of virulence in remaining strains | (Ho *et al*. 2011) |
| K7 | unknown | *wcaJ* | Intranasal infection | Lungs | Virulence was attenuated  Susceptible to phagocytosis by macrophages | (Liu, Han, and Gu 2019) |
| KPPR1 | K2 | *cpsB* | Intranasal infection | Lungs, trachea, spleen | Virulence was attenuated | (Lawlor *et al*. 2005) |
| STL43 | K1 | *wbbO* | Intraperitoneal injection | Systemic infection, liver | Attenuated | (Yeh *et al*. 2016) |
| NTUH-2044 | K1 | p-*rmpA* | Intragastric inoculation | Organ not specified, but the result is systemic infection resulting in death | Attenuated | (Hsu *et al*. 2011) |
| CG43 | K2 | p-*rmpA* | Intraperitoneal injection | Systemic infection | Attenuated | (Cheng *et al*. 2010) |
| KPPR1S | K2 | *rmpC*  *rmpA*  *rcsB* | Intranasal infection | Lungs, Spleen | Virulence was attenuated | (Walker *et al*. 2019) |
| KPPR1S  NTUH-2044 | K2  K1 | *kvrA*  *kvrB* | Intranasal infection | Lungs, Spleen | Attenuated  Attenuated | (Palacios *et al*. 2018) |
| MKP103 | Not specified | *kvrA*  *kvrB* | Intranasal infection | Lungs, Spleen | No loss of virulence  Attenuated | (Palacios *et al*. 2018) |
| STL43 | K1 | *wzy*  *rfbP*  *wcaG* | Intraperitoneal injection | Systemic infection, organs not specified | Wildtype strain killed mice in 3 days, but mutants were nonlethal at a similar dosage indicating loss of virulence. No actual graphs are plotted but results are summarised in text. | (Yeh *et al*. 2010) |

**References**

Cheng, H Y et al. 2010. “RmpA Regulation of Capsular Polysaccharide Biosynthesis in *Klebsiella pneumoniae* CG43.” *Journal of Bacteriology* 192(12): 3144–58.

Favre-Bonté, S, Tine, RL, Forestier,C. and Krogfelt, KA. 1999. “*Klebsiella pneumoniae* Capsule Expression Is Necessary for Colonization of Large Intestines of Streptomycin-Treated Mice.” *Infection and Immunity* 67(11): 6152–56.

Fung, C-P. et al. 2011. “Immune Response and Pathophysiological Features of *Klebsiella pneumoniae* Liver Abscesses in an Animal Model.” *Laboratory Investigation* 91(7): 1029–39.

Ho, J-Y. et al. 2011. “Functions of Some Capsular Polysaccharide Biosynthetic Genes in *Klebsiella pneumoniae* NTUH K-2044.” *PLoS ONE* 6(7): 1–9.

Hsu, CR et al. 2011. “The Role of *Klebsiella pneumoniae* RmpA in Capsular Polysaccharide Synthesis and Virulence Revisited.” *Microbiology* 157(12): 3446–57.

Lawlor, MS, Hsu,J, Rick, PD and Miller, VL. 2005. “Identification of *Klebsiella pneumoniae* Virulence Determinants Using an Intranasal Infection Model.” *Molecular Microbiology* 58(4): 1054–73.

Lin, C-L et al. 2017. “Effect in Virulence of Switching Conserved Homologous Capsular Polysaccharide Genes from *Klebsiella pneumoniae* Serotype K1 into K20.” *Virulence* 8(5): 487–93.

Liu, X, Han,W and Gu, J. 2019. “Three Capsular Polysaccharide Synthesis Related Glucosyltransferases, GT-1, GT-2 and WcaJ , Are Associated With Virulence and Phage Sensitivity of *Klebsiella pneumoniae*.” *Frontiers in Microbiology* 10(5): 1–14.

Maroncle, N, Balestrino,D, Rich,C, and Forestier, C. 2002. “Identification of *Klebsiella pneumoniae* Genes Involved in Intestinal Colonization and Adhesion Using Signature-Tagged Mutagenesis.” *Infection and Immunity* 70(8): 4729–34.

Palacios, M et al. 2018. “Idenfication of Two Regulators of Virulence That Are Conserved in *Klebsiella pneumoniae* Classical and Hypervirulent Strains.” *mBio* 9(4): e01443-18.

Struve, C, and Krogfelt, KA. 2003. “Role of Capsule in *Klebsiella pneumoniae* Virulence: Lack of Correlation between in Vitro and in Vivo Studies.” *FEMS Microbiology Letters* 218(1): 149–54.

Walker, KA et al. 2019. “A *Klebsiella pneumoniae* Regulatory Mutant Has Reduced Capsule Expression but Retains Hypermucoviscosity.” *mBio* 10(2): e00089-19.

Yeh, K-m. et al. 2007. “Capsular Serotype K1 or K2 , Rather than MagA and RmpA , Is a Major Virulence Determinant for *Klebsiella pneumoniae* Liver Abscess in Singapore and Taiwan.” *Journal of Clinical Microbiology* 45(2): 466–71.

Yeh, K-m. 2010. “Revisiting the Importance of Virulence Determinant MagA and Its Surrounding Genes in Klebsiella Pneumoniae Causing Pyogenic Liver Abscesses : Exact Role in Serotype K1 Capsule Formation.” *Journal of Infectious diseases* 201: 1259–67.

Yeh, K-m. et al. 2016. “Surface Antigens Contribute Differently to the Pathophysiological Features in Serotype K1 and K2 *Klebsiella pneumoniae* Strains Isolated from Liver Abscesses.” *Gut Pathogens*: 1–9.
